# Syringomyelia.—II
*"Brain," pt. li.


**Published:** 1891-06-20

**Authors:** 


### THE PRACTITIONER'S MIRROR AND RETROSPECT.

[*The Editor will be glad to receive offers of co-operation and contributions from members of the profession. All letters should be addressed to* THE EDITOR, THE LODGE, PORCHESTER SQUARE, LONDON, W.]

### MEDICINE.

### SYRINGOMYELIA.*—II.

M. Bloc adopts the following definition of the disease: Syringomyelia is a chronic affection of the spinal cord, characterised anatomically by cavities formed pathologically in this organ, and clinically by certain alterations in the sensibility, associated with trophic disorders. The pathological anatomy comprises a special principal lesion, with commonly secondary lesions; the former being situated in the spinal cord, the latter being trophic alterations in muscle, bone, cellular tissue, skin, &c. The alterations in the peripheral nerves have been carefully observed by M. Holschewnikoff and Déjerine. After opening the dura mater the cord usually presents an abnormal appearance. In a well-marked case it presents the appearance of a large blood-vessel, empty and collapsed. In ordinary cases the cord is only irregularly increased in size and deformed in places. On palpation an alteration in the consistence of the cord can easily be detected; it is soft, and there is well-marked fluctuation. If, on the other hand, the cavity is very small, one feels a hard firm cord giving the sensation of a rigid stake imbedded in the cord. On transverse section one or more characteristic cavities are visible to the naked eye. Usually only one cavity exists, but there may be two or three. These lacunæ are always situated in the grey matter—an important point to notice—their position here in order of frequence being, according to Mdlle. Baümber, the two posterior horns, the two anterior horns and any one of the four horns indifferently. The cutaneous nerves had undergone changes in those parts of the skin where the pheuomena of dissociated sensibility existed. With regard to the pathogenesis of this process, M. Brühl has very accurately pointed out the various theories without adopting any one of them. They may be summarized as follows: (*a*) That the origin of the cavity is vascular; it is a question either of œdema of the tissue around the central canal, or of hæmorrhage with subsequent reabsorption, or of colloid degeneration of the vessels. (*b*) That it is an inflammation, a myelitis. The cavity is then formed by the contraction of the sclerosed periependymal tissue, the destructive tendency of the hyperplasia, or by softening and breaking down consequent upon thrombosis of the vessels in the inflamed area. (*c*) The degeneration of the neoplasm, the glioma, is the sole cause.

But as regards the origin of this tissue of new formation, M. Bloc endorses the hypothesis of Schültze, which reconciles the two distinct views held by many observers, viz., on the one hand that it is the production of an inflammatory hyperplasia, on the other that it is a neoplastic hyperplasia. M. Bloc is led to regard the process as a slow proliferation of the neuroglia, resulting in the formation of the so-called gliomatous tissue. This tissue, as it gives rise to a number of tissues of pathological origin, would soon present phenomena of disintegration, either from disorders of the circulation, or from the simple fact of the natural tendencies of the tissue, and a cavity would thus make its appearance.

Very little is known about the causes of syringomyelia. The age at which it first begins to show itself varies between fifteen and thirty-five years. Statistics show that men are attacked more frequently than women, in the proportion of three. We have collected but few references to any hereditary nervous tendencies. Injury, overwork, chills, infectious diseases, drink, appear to be determining causes, but sometimes none of these influences are discoverable in the previous history of the patients. Consequently one is disposed to think that the cause lies in some embryological defect, upon which the chance causes we have mentioned might have had but an accidental effect.

The symptoms of syringomyelia vary greatly, but a mean type may be described, which will give a general idea of the condition. Thus the disease may be found to commence insidiously, generally by increasing weakness in the upper limbs, together with numbness in these limbs. Soon muscular atrophy is added to the functional weakness. Sensory disorders become more marked. Spinal curvature in the form of scoliosis becomes a symptom, and finally various motor troubles are added. Examination of the patient at this point shows the following points: The attitude is peculiar, due to the curvature of the spinal column. There is a claw-like deformity of the hands, similar to that seen in progressive muscular atrophy. The hands are wasted, and present trophic changes, such as ulcers and painless whitlows. There is noticeable loss of muscular power, and the tendon-reflexes are absent or diminished. Sensibility to touch is retained, but sensibility to pricking, as well as to heat and cold, are abolished, not only over the upper extremities, but over more or less extensive areas elsewhere.

The most important clinical factor in the diagnosis is the pathological dissociation of the different kinds of sensibility. The law is, although it is by no means absolute, that three forms of sensibility affected in the disease, viz., to pain, heat, and cold. Three forms are unaffected, viz., sensibility to touch, the muscular sense, and the special senses. Trenno-anæsthesia is often overlooked. It is necessary to distinguish sensibility to heat from that of cold, for the areas do not always correspond.

Except so far as appertains to the permanent paralysis of the upper extremities, the disorders of motion are only secondary. They consist of spasmodic paraplegia, rarely complete, or of inco-ordination of the lower limbs. The patellar reflexes may be exaggerated or abolished. Scoliosis, which is almost a constant feature of syringomyelia, makes its appearance at a relatively early age. It attacks the dorsi-lumbar region, and has its convexity to the left.

Of the trophic changes, the most constant are the muscular atrophy. It commences in the hands, attacking the muscles supplied by the radial nerve, or those supplied by the median and ulnar. The atrophy afterwards extends slowly and symmetrically to the forearm, arm, and trunk, resulting in deformities like those caused by other progressive myopathies. The skin is often affected with glossiness, and with eruptions, such as bullæ, herpes, etc. The subcutaneous cellular tissue is sometimes œdematous; in others abscesses and whitlows are present. Vascular disorders are of common occurrence, and the extremities are often cold and cyanosed. The joints are sometimes the seat of arthropathy like those of tabes, and the bones may be thickened, brittle, or the seat of exostoses.

The lesions of syringomyelia may extend to the bulb, and, consequently, bring about disorders of deglutition, of the circulation, and of respiration. This condition is comparatively rare, usually the digestive and cardio-pulmonary systems are intact. Inequality of the pupils may be observed, caused by the presence of a cilio-spinal centre at the level of the cervico-dorsal enlargement of the cord. The bladder generally shows evidences of cystitis which appear to be due to an interference with the nutrition of the bladder rather than to disturbances of the vesico-spinal centre.

* "Brain," pt. li.

Various other forms of this disease occur, and M. Bloc thinks two principal types may be distinguished depending on the localisation of the neoplasm. In one of these forms, it commences with an atrophy of the muscles supplied by the ulnar nerve, and is accompanied by spasmodic phenomena of the lower limbs. The other begins by an atrophy of the muscles supplied by the radial, and is accompanied by tabetic symptoms in the lower limbs.

The disease progresses slowly, and the cause is varied by exacerbations and remissions, with an invariable downward tendency ending in death, which may be due to secondary complications, or bulbar complications may supervene.

The treatment can be scarcely more than symptomatic, and the atrophy of the muscles may be combated by electricity. Counter-irritation to the spine may be tried.

